# Supplementary material for: In Silico Analysis of the Structural Dynamics and Substrate Recognition Determinants of the Human Mitochondrial Carnitine/Acylcarnitine SLC25A20 Transporter
Source: Int J Mol Sci. 2023 Feb 15;24(4):3946. doi: 10.3390/ijms24043946 (PMC9961348; doi:10.3390/ijms24043946)
Supplement: Supplementary file 1 [file ijms-24-03946-s001.zip › ijms-2147358-supplementary.docx]

*Supplementary Materials*

*In silico* analysis of the structural dynamics and substrate recognition determinants of the human mitochondrial carnitine/acylcarnitine SLC25A20 transporter.

Andrea Pasquadibisceglie^1†^, Virginia Quadrotta^1†^ and Fabio Polticelli ^1,2,^*

^1^ Department of Sciences, University of Roma Tre, Rome, Italy; andrea.pasquadibisceglie@uniroma3.it, vir.quadrotta@stud.uniroma3.it, fabio.polticelli@uniroma3.it

^2^ National Institute of Nuclear Physics, Roma Tre Section, Rome, Italy; fabio.polticelli@uniroma3.it

***** Correspondence: fabio.polticelli@uniroma3.it;

^†^ Authors contributed equally to this work

**Table S1.** Analysis of the hydrogen bonds formed by the PCAR ligand with protein residues during the c-state simulations (for each pair, only the most frequent interaction is reported and only those with a persistence higher than the 10% of the trajectory are shown).

|  | **MD1** |  |
| --- | --- | --- |
| **Acceptor** | **Donor** | **Fraction** |
| PCAR@O10 | TYR_190@OH | 0.389 |
| PCAR@O11 | TYR_190@OH | 0.254 |

|  | **MD2** |  |
| --- | --- | --- |
| **Acceptor** | **Donor** | **Fraction** |
| PCAR@O10 | TYR_190@OH | 0.329 |
| PCAR@O11 | TYR_190@OH | 0.305 |
| PCAR@O10 | TYR_186@OH | 0.123 |

**Table S2.** Analysis of the salt bridge interactions formed by the PCAR ligand with protein residues during the c-state simulations (for each pair, only the most frequent interaction is reported and only those with a persistence higher than the 10% of the trajectory are shown).

|  | **MD1** |  |
| --- | --- | --- |
| **Acceptor** | **Donor** | **Fraction** |
| PCAR@O11 | LYS_194@NZ | 0.127 |
| PCAR@O10 | LYS_194@NZ | 0.119 |

|  | **MD2** | |  | |  |
| --- | --- | --- | --- | --- | --- |
| **Acceptor** | | **Donor** | | **Fraction** | |
| PCAR@O10 | | LYS_194@NZ | | 0.133 | |
| PCAR@O11 | | LYS_194@NZ | | 0.129 | |
|  | |  | |  | |

**Table S3.** Analysis of the hydrophobic interaction formed by the PCAR ligand with protein residues during the c-state simulations (for each pair, only the most frequent interaction is reported and only those with a persistence higher than the 5% of the trajectory are shown).

| **MD1** | |  | **MD2** | |
| --- | --- | --- | --- | --- |
| **Contact** | **Fraction** |  | **Contact** | **Fraction** |
| PCAR@C16_:287@CG | 0.166 |  | PCAR@C2_:190@CZ | 0.215 |
| PCAR@C8_:190@CE1 | 0.104 |  | PCAR@C15_:187@CZ | 0.118 |
| PCAR@C14_:86@CD1 | 0.088 |  | PCAR@C8_:287@CD2 | 0.1 |
| PCAR@C1_:90@CD2 | 0.077 |  | PCAR@C16_:186@CE1 | 0.087 |
| PCAR@C1_:187@CZ | 0.076 |  | PCAR@C1_:86@CE2 | 0.076 |
| PCAR@C1_:93@CD1 | 0.063 |  | PCAR@C8_:24@CD1 | 0.062 |
| PCAR@C3_:212@CG2 | 0.057 |  | PCAR@C1_:284@CB | 0.05 |

**Table S4.** Analysis of the hydrogen bonds formed by the CAR ligand with protein residues during the m-state simulations.

|  | **MD1** |  |
| --- | --- | --- |
| **Acceptor** | **Donor** | **Fraction** |
| CAR@O12 | TRP_224@NE1 | 0.29 |
| CAR@O11 | TRP_224@NE1 | 0.1765 |
| CAR@O12 | ASN_280@ND2 | 0.173 |
| CAR@O11 | ASN_280@ND2 | 0.1335 |

|  | **MD2** |  |
| --- | --- | --- |
| **Acceptor** | **Donor** | **Fraction** |
| CAR@O11 | TRP_224@NE1 | 0.842 |
| CAR@O11 | ASN_280@ND2 | 0.514 |
| TYR_186@OH | CAR@O8 | 0.119 |

**Table S5.** Analysis of the hydrophobic interactions formed by the CAR ligand with protein residues during the m-state simulations (for each pair, only the most frequent interaction is reported and only those with a persistence higher than the 5% of the trajectory are shown).

| **MD1** | |  | **MD2** | |
| --- | --- | --- | --- | --- |
| **Contact** | **Fraction** |  | **Contact** | **Fraction** |
| CAR@C4_:224@CZ2 | 0.474 |  | CAR@C3_:224@CE2 | 0.796 |
| CAR@C1_:227@CB | 0.143 |  | CAR@C6_:86@CZ | 0.257 |
| CAR@C9_:24@CD1 | 0.139 |  | CAR@C9_:24@CD1 | 0.203 |
| CAR@C9_:86@CZ | 0.125 |  | CAR@C3_:227@CB | 0.153 |

**Table S6. Representative residues composition of the apo and holo protein-solvent-membrane systems.** The residue names are in accordance with the CHARMM36m force field. For the detailed composition of each simulated system see the relative file parameters in the uploaded dataset (see Data Availability statement).

| **Apo** | | **Holo** | |
| --- | --- | --- | --- |
| **Residue name** | **Number of molecules** | **Residues name** | **Number of molecules** |
| **Water** | | | |
| WAT | 21217 | WAT | 20901 |
| **Ions** | | | |
| SOD | 97 | SOD | 96 |
| CLA | 56 | CLA | 56 |
| **Lipids** | | | |
| POPC | 10 | POPC | 13 |
| PLPC | 29 | PLPC | 29 |
| SAPC | 48 | SAPC | 48 |
| POPE | 9 | POPE | 9 |
| PLPE | 16 | PLPE | 16 |
| SAPE | 48 | SAPE | 48 |
| SAPS | 6 | SAPS | 6 |
| SAPI | 11 | SAPI | 11 |
| TLCL | 35 | TLCL | 35 |
| **Ligand** | | | |
|  |  | CAR/PCAR | 1 |


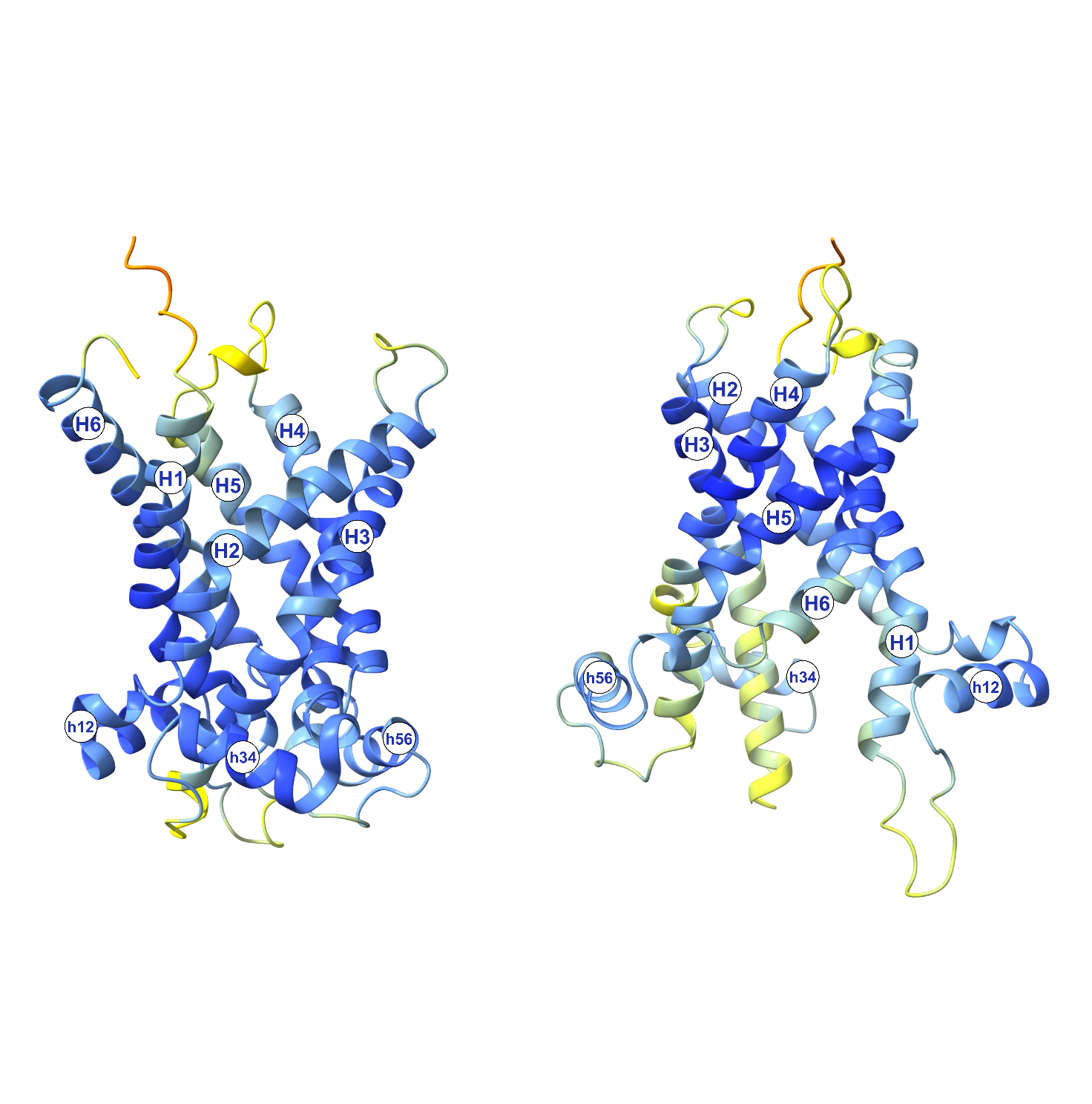


**Figure S1. Three-dimensional structural models of the human SLC25A20 c- and m-state.** Three-dimensional models of the human SLC25A20 c- (left) and m-state (right), obtained with a custom implementation of AlphaFold2 (see Methods section), depicted with ribbon representation and colored by pLDDT score.


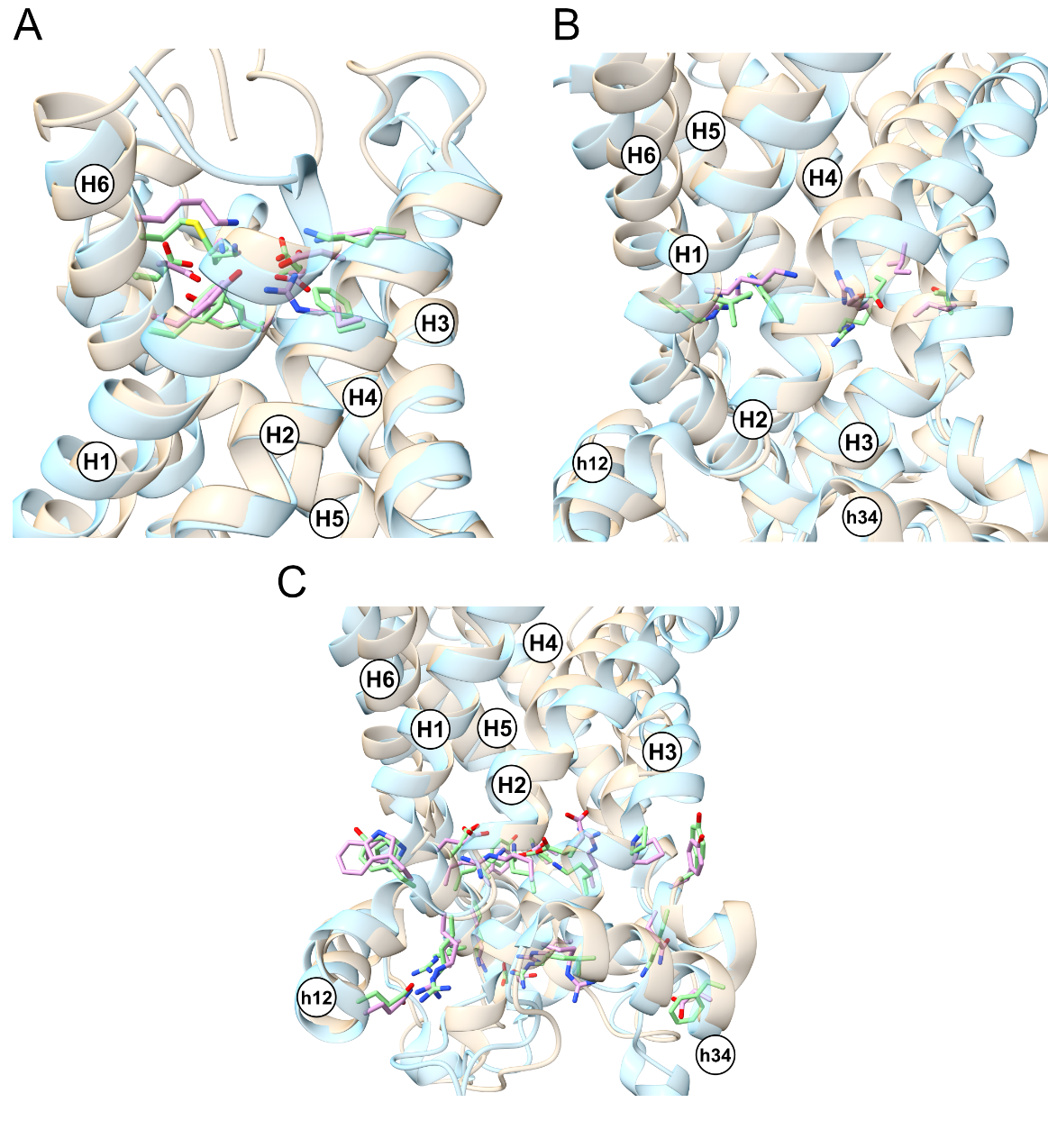


**Figure S2. Superimposition between the human SLC25A20 structural model and the fungal AAC crystal structures.** (A) Superimposition between the m-state structural model of the human SLC25A20 and the crystal structure of the AAC from *Thermothelomyces thermophilus* (PDB ID: 6GCI). Residues of the cytoplasmic [FY][DE]XX[KR] motif are shown as sticks (SLC25A20 in green; AAC in pink); (B, C) Superimposition between the c-state structural model of the human SLC25A20 and the crystal structure of the AAC from *Bos taurus* (PDB ID: 1OKC). Residues of the central binding site (B) and residues of the motif PX[DE]XX[KR]X[KR]X_20-30_[DE]GXXXX[WYF][KR]G (C) are shown as sticks (SLC25A20 in green; AAC in pink).

**
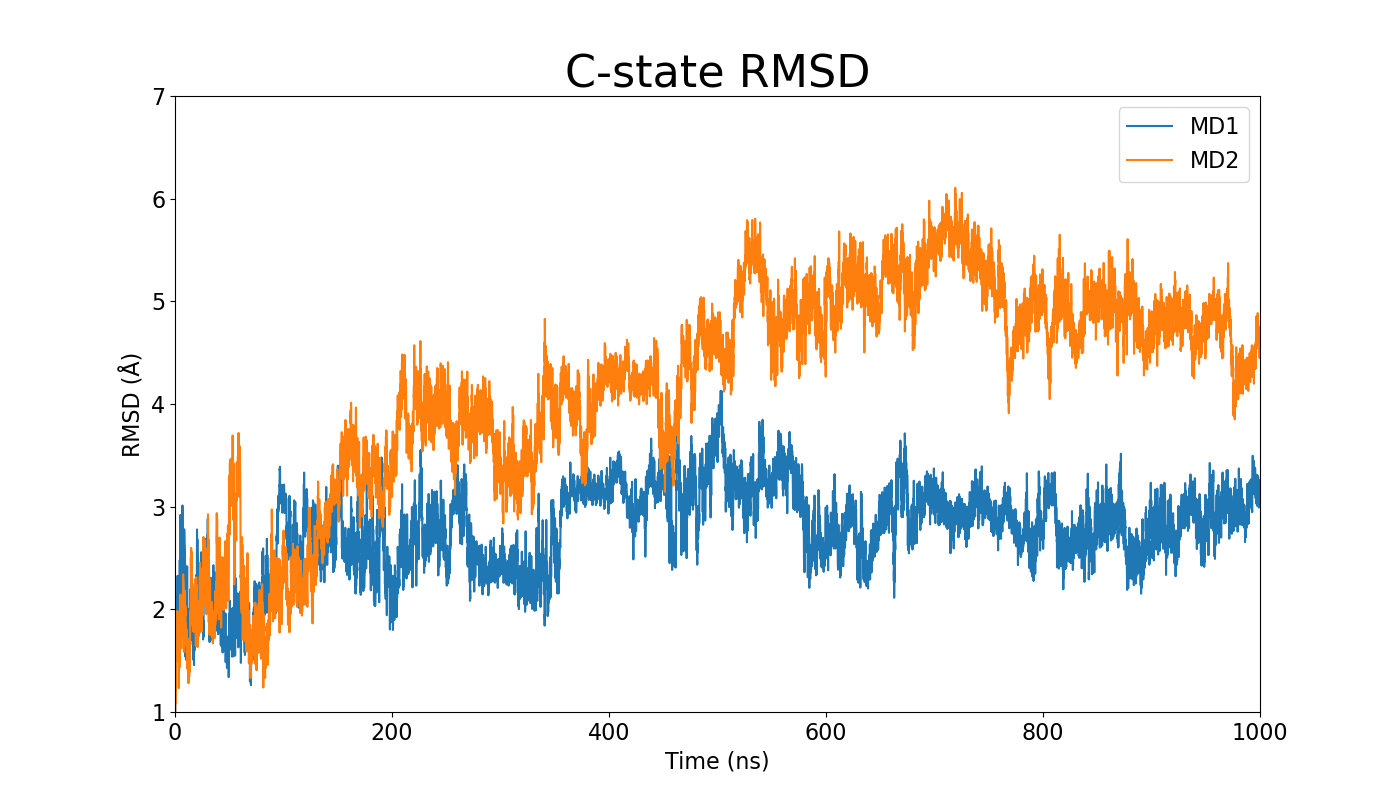
**

**Figure S3. RMSD time series of the SLC25A20 c-state simulations.**

**
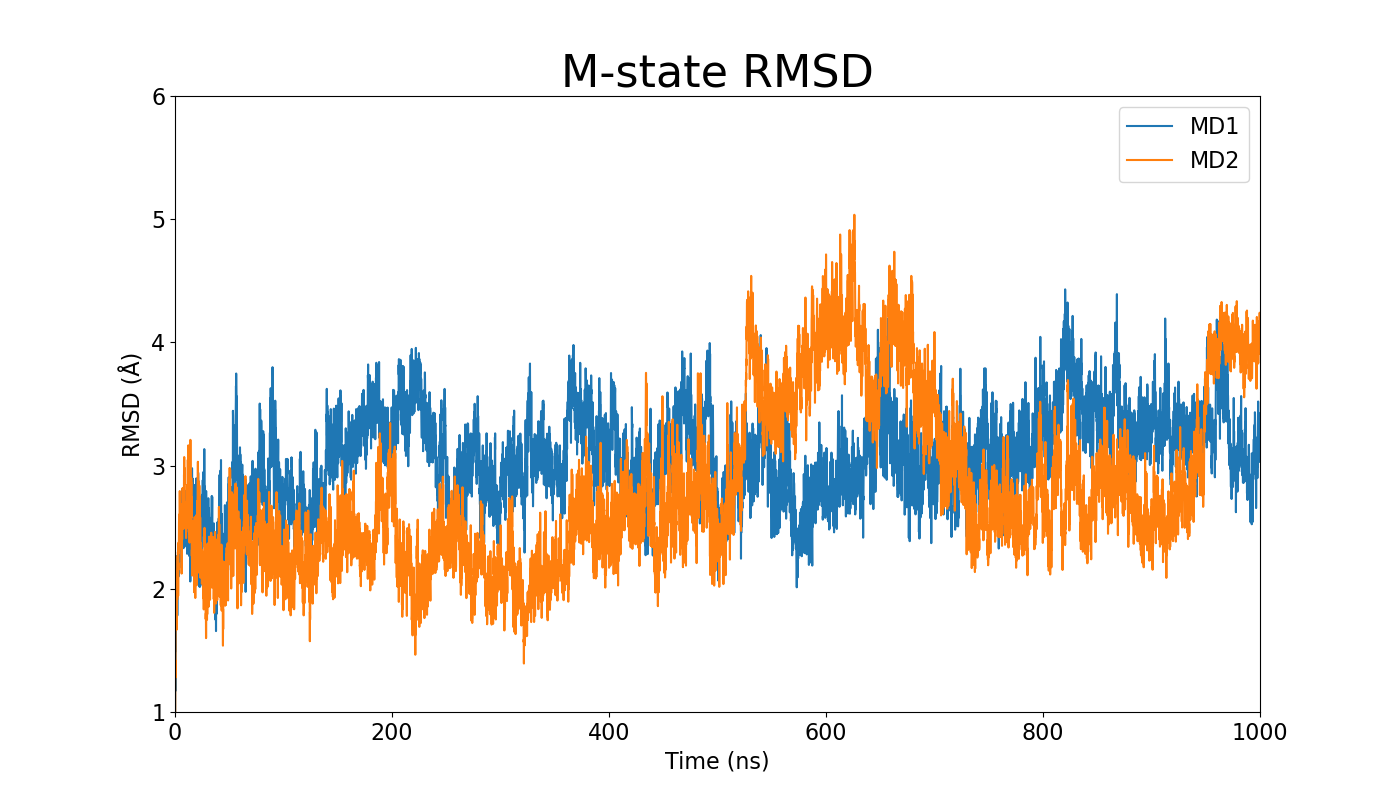
**

**Figure S4. RMSD time series of the SLC25A20 m-state simulations.**

**
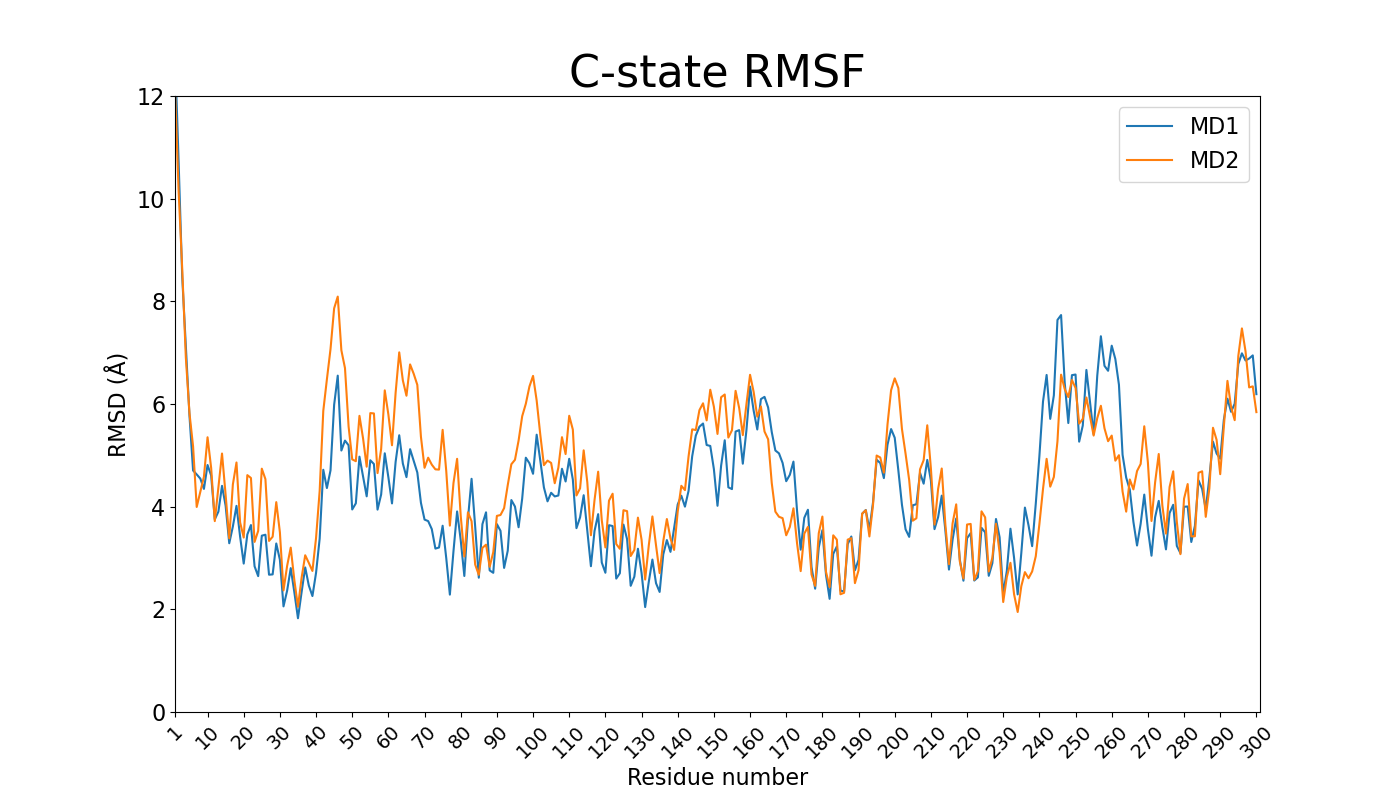
**

**Figure S5. RMSF analysis of the SLC25A20 c-state simulations.**

**
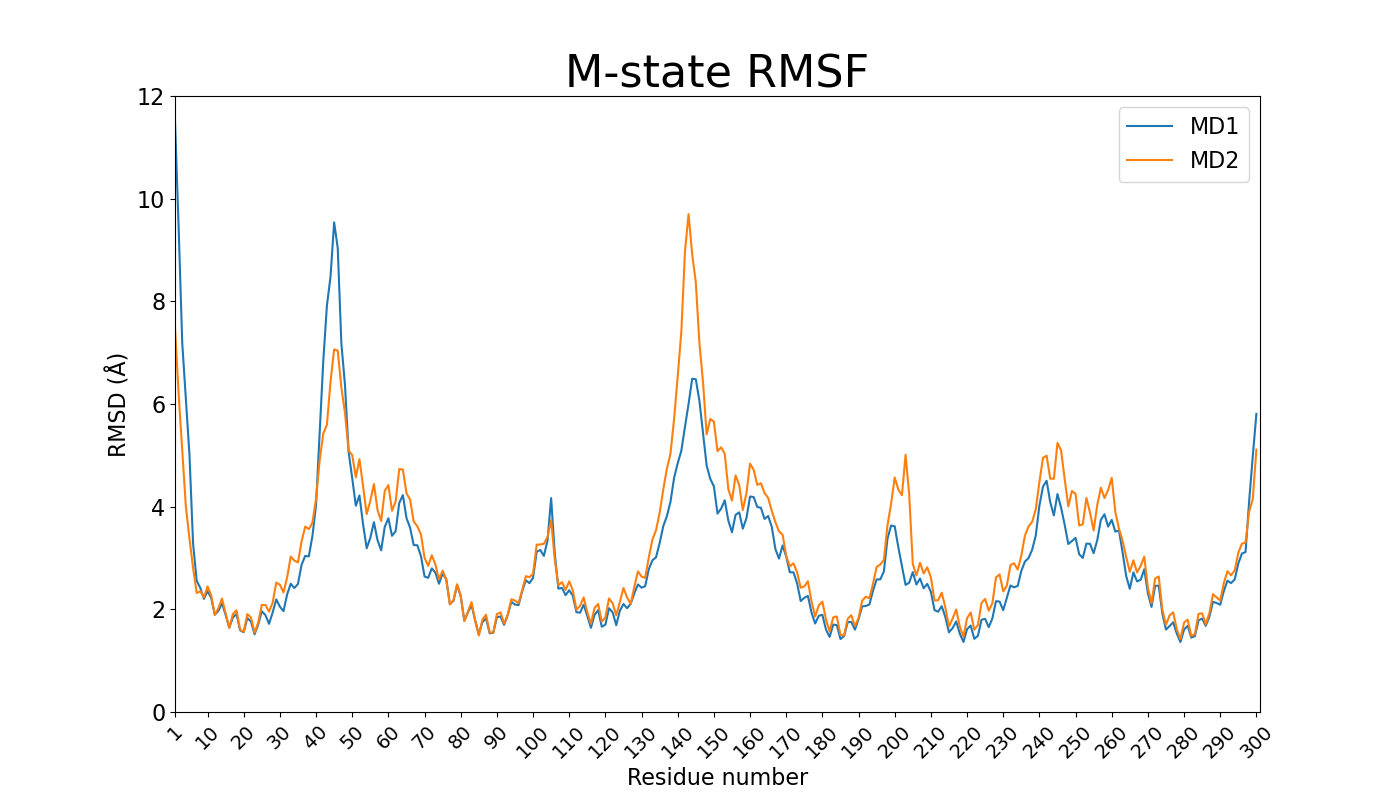
**

**Figure S6. RMSF analysis of the SLC25A20 m-state simulations.**

**
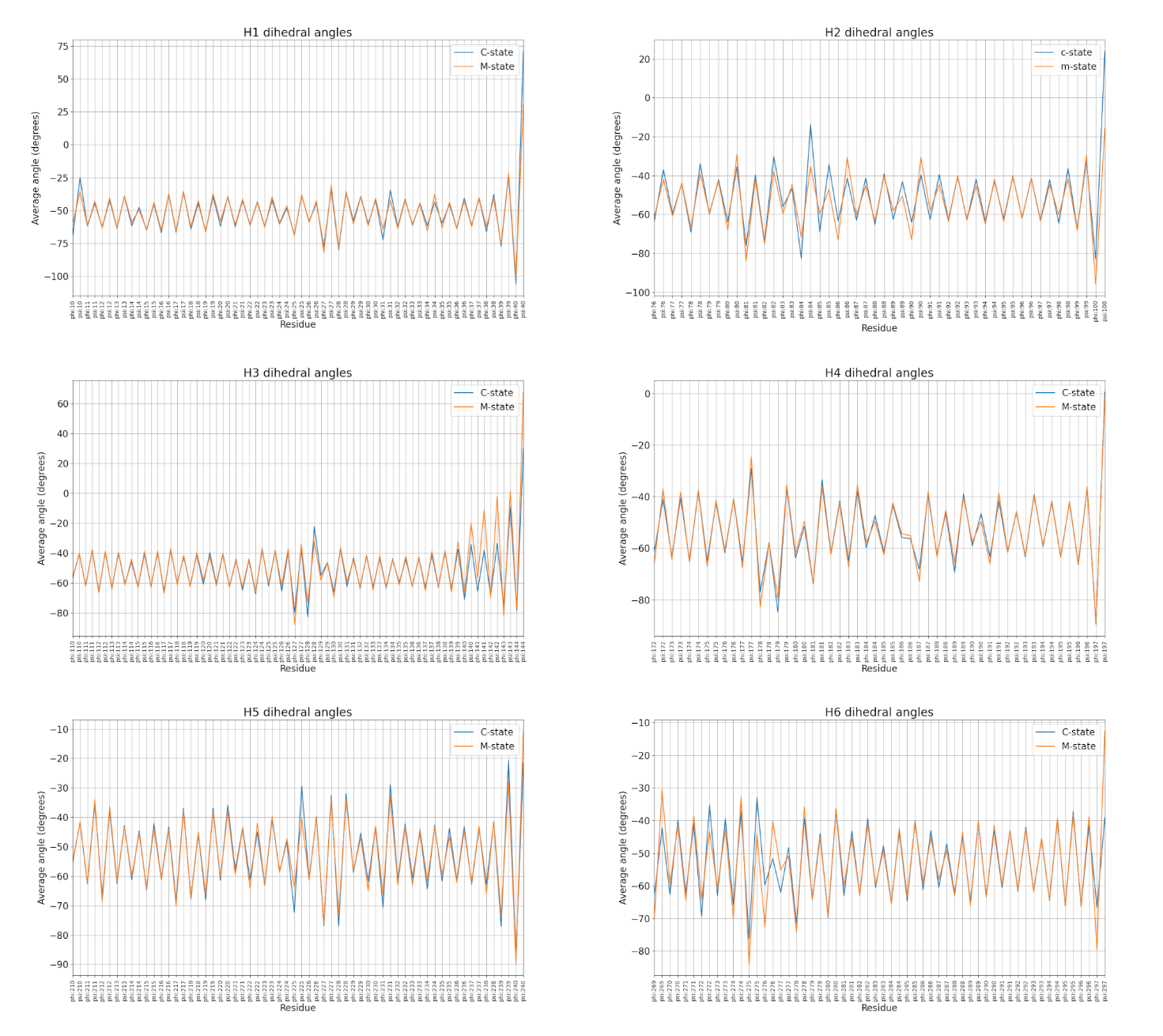
**

**Figure S7. Transmembrane helices backbone dihedral angles analysis.**

**
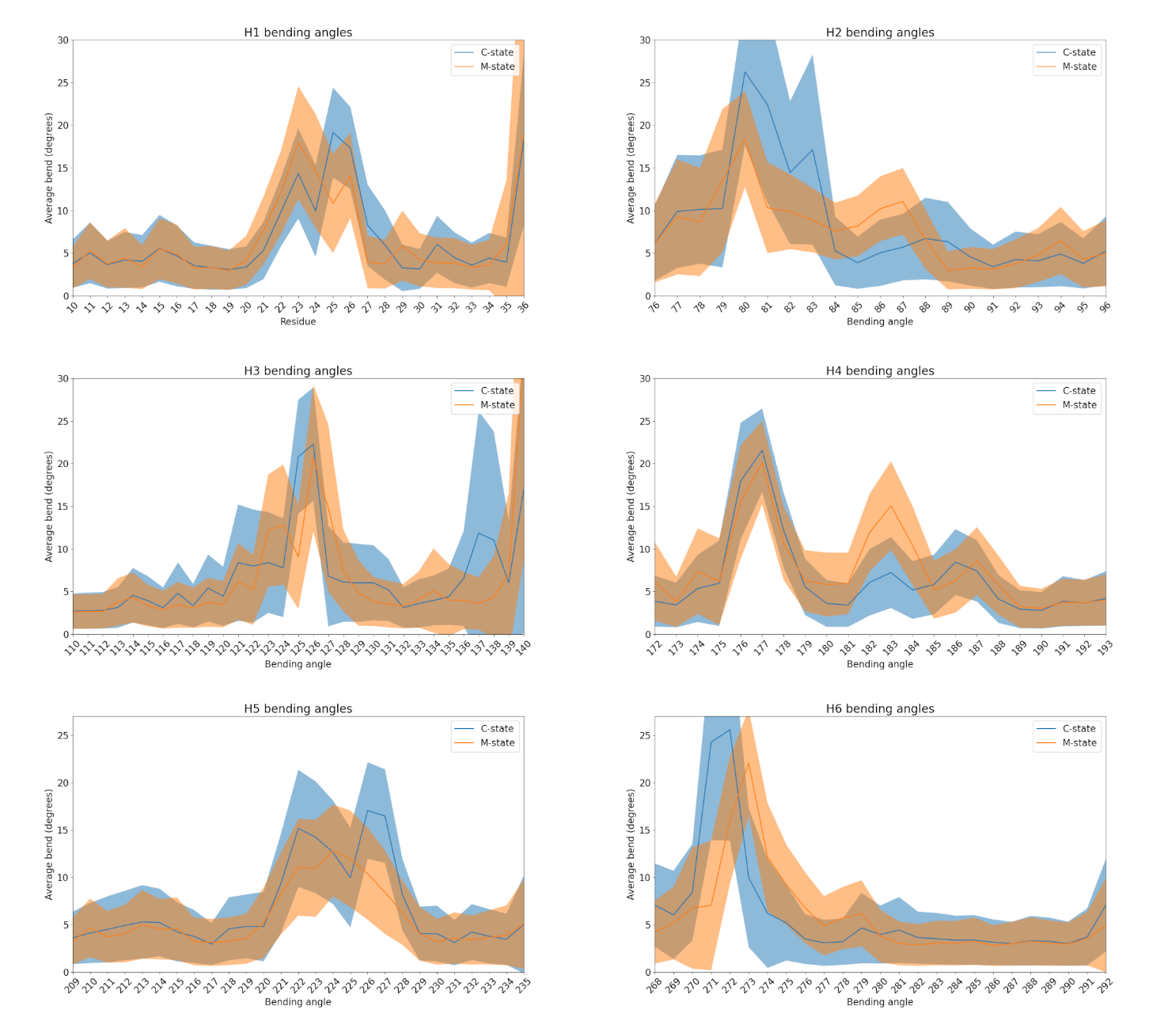
**

**Figure S8. Transmembrane helices bending angles analysis.**


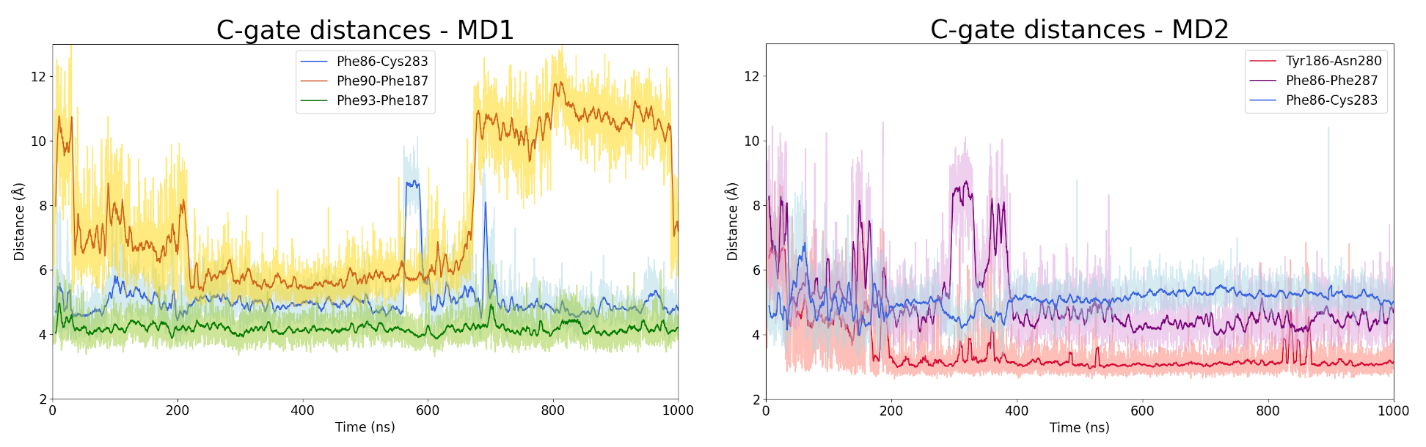


**Figure S9. Distances between cytoplasmic gate residues side-chains along the c-state MD trajectories.**

**
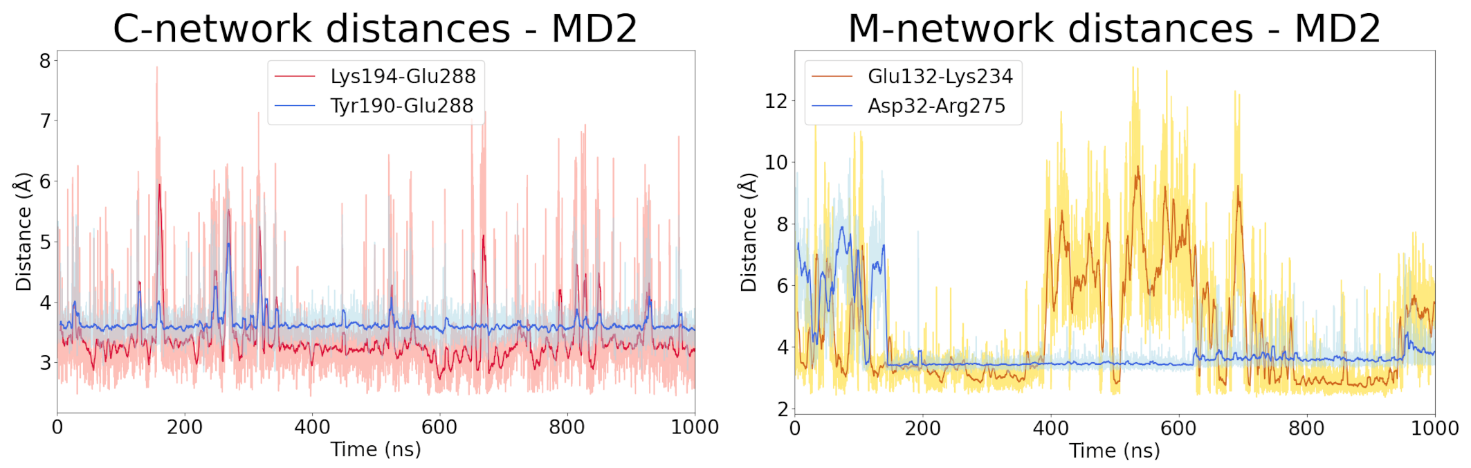
**

**Figure S10. Cytoplasmic and matrix network residues distances along the m-state MD2 trajectory**

**
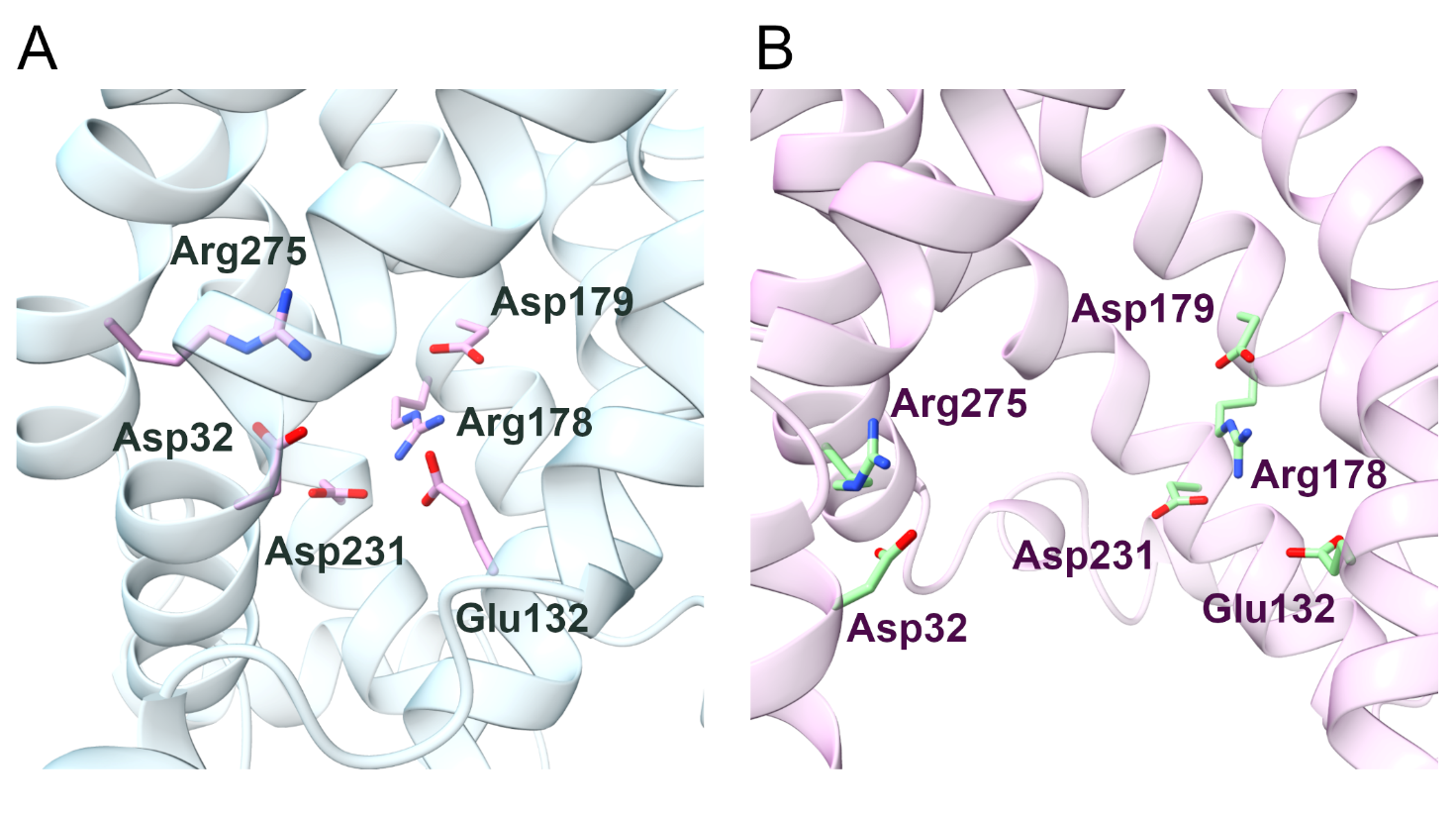
**

**Figure S11. Representative conformations of the contact points interacting residues**

**
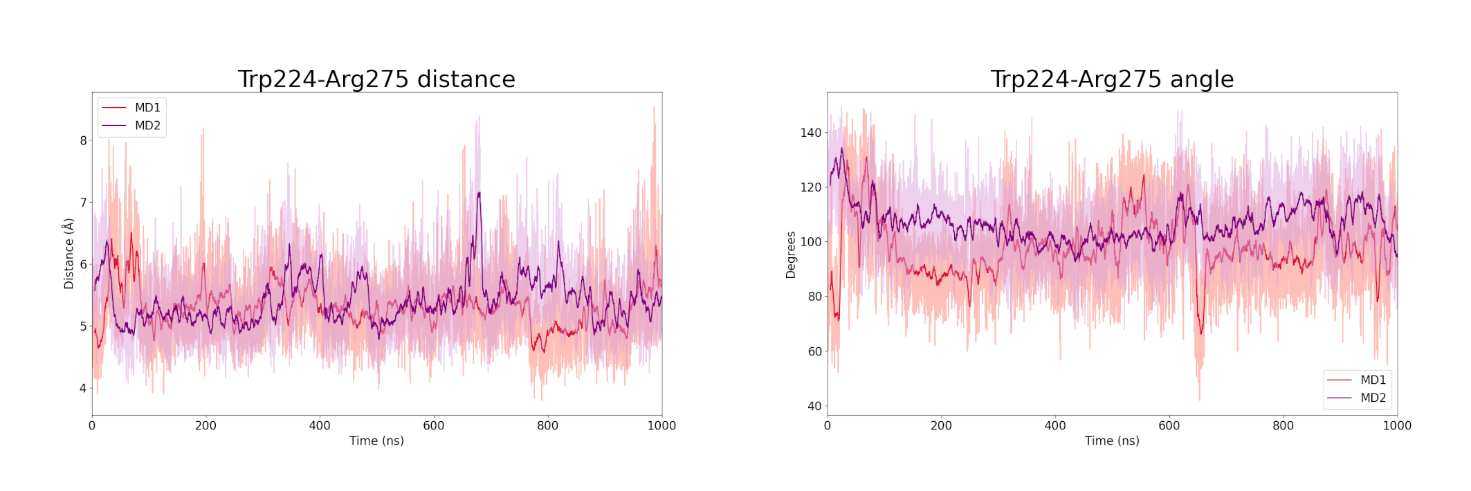
Figure S12. Distances and angles between Trp224 and Arg275**

**
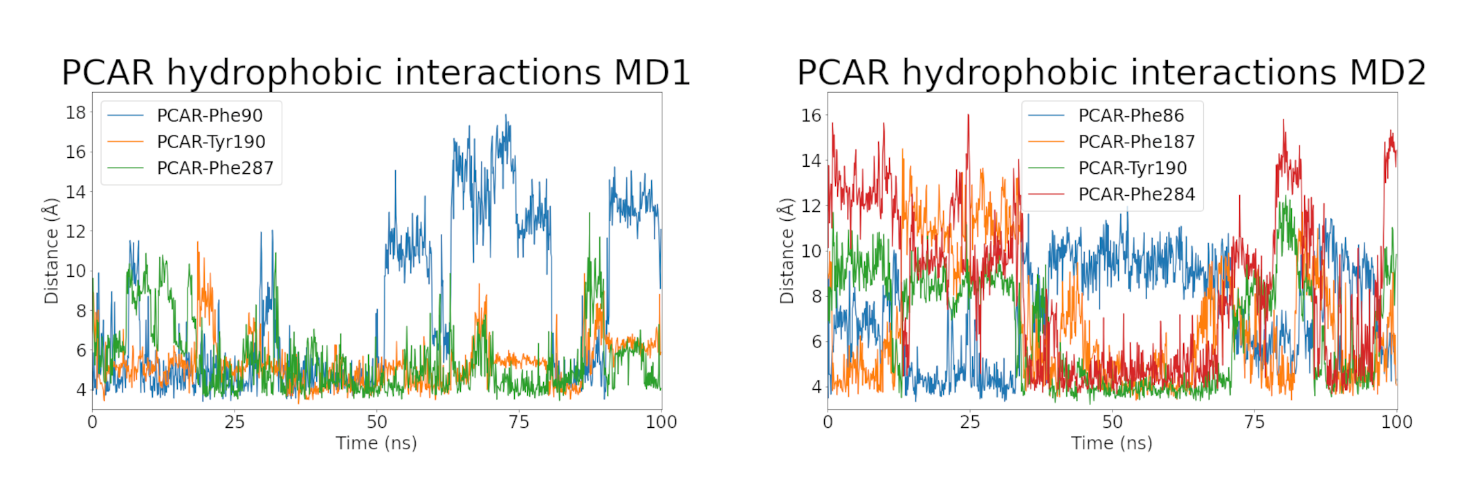
Figure S13. SLC25A20-PCAR hydrophobic contact distances**


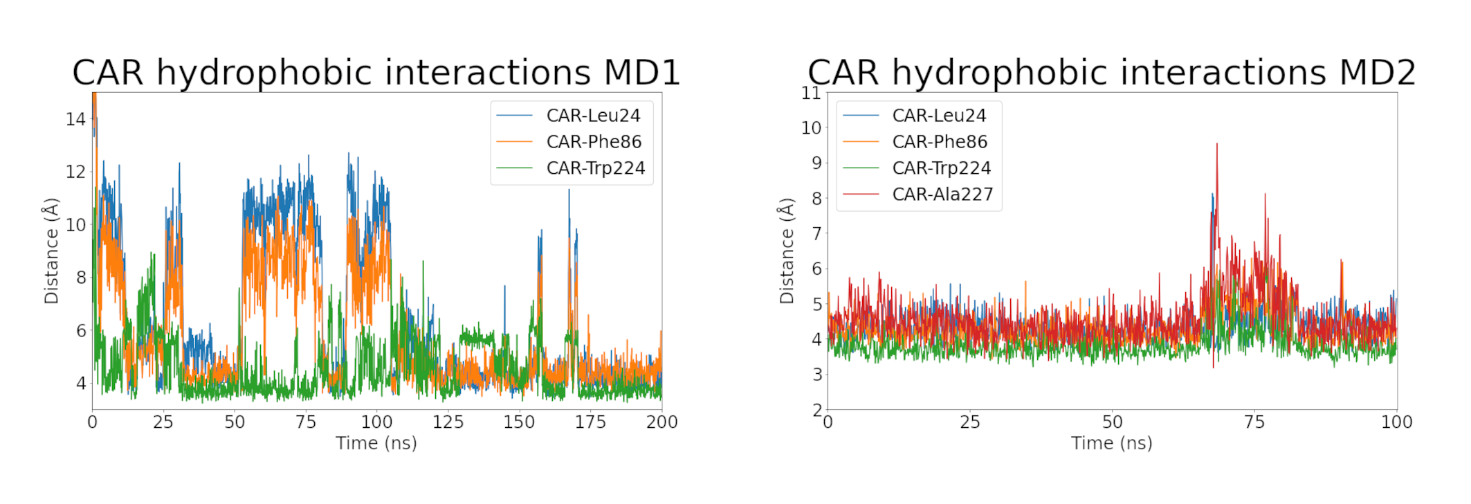
**Figure S14. SLC25A20-CAR hydrophobic contact distances**
